# Association between Changes in Plasma Metabolism and Clinical Outcomes of Sepsis

**DOI:** 10.1155/2023/2590115

**Published:** 2023-06-13

**Authors:** Xin Li, Zhongnan Yin, Wei Yan, Meng Wang, Chun Chang, Chenglin Guo, Lixiang Xue, Qingtao Zhou, Yongchang Sun

**Affiliations:** ^1^Department of Respiratory and Critical Care Medicine, Peking University Third Hospital, 49 North Garden Road, Haidian District, Beijing 100191, China; ^2^Institute of Medical Innovation and Research, Peking University Third Hospital, Beijing 100191, China; ^3^Biobank, Peking University Third Hospital, Beijing 100191, China

## Abstract

Current prognostic biomarkers for sepsis have limited sensitivity and specificity. This study aimed to investigate dynamic lipid metabolomics and their association with septic immune response and clinical outcomes of sepsis. This prospective cohort study included patients with sepsis who met the Sepsis 3.0 criteria. On hospitalization days 1 (D1) and 7 (D7), plasma samples were collected, and patients underwent liquid chromatography with tandem mass spectrometry. A total of 40 patients were enrolled in the study, 24 (60%) of whom were men. The median age of the enrolled patients was 81 (68–84) years. Thirty-one (77.5%) patients had a primary infection site of the lung. Participants were allocated to the survivor (25 cases) and nonsurvivor (15 cases) groups based on their 28-day survival status. Ultimately, a total of 113 lipids were detected in plasma samples on D 1 and D 7, of which 42 lipids were most abundant in plasma samples. The nonsurvival group had significantly lower lipid expression levels in lysophosphatidylcholine (LysoPC) (16 : 0, 17 : 0,18 : 0) and 18 : 1 SM than those in the survival group (*p* <  0.05) on D7–D1. The correlation analysis showed that D7–D1 16 : 0 LysoPC (*r* = 0.367, *p* = 0.036),17 : 0 LysoPC (*r* = 0.389, *p* = 0.025) and 18 : 0 LysoPC(*r* = 0.472, *p* = 0.006) levels were positively correlated with the percentage of CD3^+^ T cell in the D7–D1. Plasma LysoPC and SM changes may serve as prognostic biomarkers for sepsis, and lipid metabolism may play a role in septic immune disturbances.

## 1. Introduction

Sepsis is an infection-induced systemic inflammatory response involving multiple mediators and cytokines. It is a commonly encountered life-threatening condition in intensive care units (ICUs) worldwide [1]. Despite medical advances, sepsis remains a serious global health problem with an estimated 48.9 million cases and 11 million deaths annually [2]. Martin et al. [3] found that over a period of 24 years (1979–2002), the rate of increase in sepsis incidence was 20.4% higher in those aged ≥65 years than that in their counterparts. The sepsis risk in 65-year-old individuals grew by an average of 9.5% per year over 24 years, and the incidence rate for people aged 65 years and older increased by 11.5% annually. Currently, the Acute Physiology and Chronic Health Evaluation (APACHE) II score is commonly used in the ICU to assess the severity of hospitalized patients [4]. However, this method has some limitations and fails to reflect prognosis in patients whose disease peak is not observed within the first 24 hours after admission.

Research interest in the association between lipid metabolism and sepsis is increasing [5, 6]. Recent studies suggest that poor prognosis is related to increased free fatty acid levels, changes in polyunsaturated fatty acid metabolism [7], decreased lysophosphatidylcholine (LysoPC) levels [8], and elevated plasma ceramide levels [9]. In addition, Park et al. [10] conducted a serum lipid analysis of 74 ICU patients and found that LysoPC levels in the survival group were higher than those in the mortality group and that LysoPC monitoring helped predict the 28-day mortality rate of patients with sepsis and septic shock. These studies suggest that lipidomic analysis may help develop prognostic markers for sepsis.

To further examine the role of lipid metabolism in sepsis, this study aimed to investigate dynamic lipid metabolomics and their association with T cells and the septic immune response and clinical outcomes of sepsis.

## 2. Materials and Methods

### 2.1. Study Design and Period

This prospective cohort study was conducted at the medical ICU (MICU) of a university-affiliated urban teaching hospital from December 2019 to February 2022. The study protocol was approved by the medical science research ethics committee of our hospital (approval no. M2019396). All patients or their legally authorized representatives provided written informed consent to participate in the study.

### 2.2. Study Population

A total of 40 patients with sepsis admitted to the MICU were included and subsequently divided into the survivor and nonsurvivor groups based on their 28-day survival status. The inclusion criteria were as follows: (1) patients who met the Sepsis 3.0 diagnostic criteria [11] and (2) were aged ≥18 years. The exclusion criteria were as follows: (1) pregnancy, (2) human immunodeficiency virus or tuberculosis infection, and (3) a “do-not-resuscitate” or discontinuation of tracheal intubation order.

### 2.3. Clinical and Laboratory Data

Data on the following variables were collected: age, sex, body weight, height, body mass index (BMI), days of mechanical ventilation, and length of stay at discharge or death. Finally, plasma (4 mL) samples were collected on day 1 (D1) and day 7 (D7) of hospitalization. The collected samples were aliquoted into multiple tubes to avoid repeated thawing and freezing. Samples were stored in a −80°C freezer until use.

### 2.4. Flow Cytometry

Whole blood was collected directly into anticoagulant tubes and centrifuged at 1,800 × *g* for 10 min. The cells were treated with ammonium-chloride-potassium lysing buffer and stained with fluorescence-labeled antibodies for 15 min in 1 × phosphate-buffered saline. All experiments were performed using the Beckman CytoFLEX S flow cytometer (Beckman Coulter, Brea, CA, USA). Kaluza Analysis 2.1 software was used for data analysis. The following antibodies were used in this experiment: CD45-allophycocyanin (clone HI30), CD3-fluorescein-5-isothiocyanate (clone HIT3a), CD4-phycoerythrin (PE) (clone RPA-T4), CD8-peridinin chlorophyll protein/cyanine 5.5 (clone SK1), and CD8-PE/Dazzle™ (clone SK1). All antibodies were purchased from BioLegend (San Diego, CA, USA).

### 2.5. Lipid Analysis

Ammonium acetate (purity ≥99%) and formic acid (purity ≥98%) were purchased from Fluka (Charlotte, NC, USA); acetonitrile, water and isopropyl alcohol from Thermo Fisher Scientific (Waltham, MA, USA); and saline from Dubang Pharmaceuticals Co., Ltd. (Changchun, China).

Samples stored at −80°C were thawed in a 4°C refrigerator. Subsequently, a 10 *µ*L sample aliquot was sequentially supplemented with 10 *µ*L of an internal standard mix solution, 10 *µ*L of water, and 100 *µ*L of chloroform: methanol (2 : 1, v/v) extracting solution. The resulting mixture was vortexed for 20 s, placed in a 4°C refrigerator for 30 min, and then centrifuged at 7,800 × *g* for 3 min. Subsequently, the supernatant was collected using a 1-mL syringe and placed into a 0.5-mL EP tube. Thereafter, 40 *µ*L of the supernatant was immediately placed into a clean EP tube and evaporated until dry via nitrogen gas blowdown. The tube was sealed and stored in a −20°C freezer until use. Before injection, the sample was dissolved in 50 *µ*L of acetonitrile and isopropyl alcohol (1 : 1, v/v) and vortexed for 60 s.

All samples underwent liquid chromatography (LC) analysis using an Ekspert UltraLC 100 System (Eksigent Technologies, Silicon Valley, CA, USA), followed by mass spectrometry analysis using an AB SCIEX TripleTOF 5600 System (Eksigent Technologies, Silicon Valley, CA, USA). The standard was 19 : 0 LysoPC, 19 : 0 phosphatidylcholine (PC), 17 : 0 phosphatidylethanolamine (PE), 12 : 0 SM, and 19 : 0 ceramide. The optimized mass spectrometer settings were as follows: gas, 50 psi; gas 2, 50 psi; curtain gas, 30 psi; ion source temperature, 550°C; ion spray voltage floating, −4,500 V; and mass range, 50−1,200 Da. An acquisition method was established in the negative ion mode to ensure the accurate identification of each analyte. The LC conditions were as follows: chromatography column, Waters (Milford, MA, USA) XBridge Peptide BEH C18 (2.1 × 100 mm, 3.5 *μ*m; Waters); mobile phase: A, 10 mM ammonium acetate + 0.1% formic acid + 99.9% water; mobile phase: B, 10 mM ammonium acetate + 0.1% formic acid + 49.95% acetonitrile + 49.95% isopropyl alcohol; operating temperature, 40°C; flow rate, 0.4 mL/min; and run time, 20 min. The gradient program for the initial solvent B was as follows: 0.01–2.00 min, 35–80% B; 2.0–9.0 min, 80–100% B; 9.0–15.0 min, 100% B; 15.0–16.0 min, 100−35% B (hold until 20.0 min for the next run; and injection volume, 2 *μ*L. Regarding the lipid standard database, the PeakView v.1.2 software (SCIEX, Framingham, MA, USA) was used to characterize the sample lipids, and MultiQuant v.2.1 (SCIEX) was used to quantify qualitative lipids.

### 2.6. Statistical Analyses

Clinical data were compared between the survivor and nonsurvivor groups. SPSS software v.21.0 (IBM Corp., Armonk, NY, USA) was used for statistical analysis. Continuous variables are expressed as medians (range), while categorical variables are expressed as frequencies (%). Continuous variables were compared using the nonparametric Mann–Whitney *U*-test, and categorical variables were compared using the *χ*^2^ test. The degree of correlation was expressed using Pearson's correlation coefficient “r.” A *p*  <  0.05 indicated statistical significance.

## 3. Results

### 3.1. Patient Characteristics

We included 40 patients with sepsis, 24 (60%) of whom were men. Participants were allocated to the survivor (*n* = 25) and nonsurvivor (*n* = 15) groups based on their 28-day survival status. The median age of the enrolled patients was 81 (68–84) years. Thirty-one (77.5%) patients had a pulmonary infection.  Table 1 shows the detailed characteristics of the participants.

### 3.2. Lipidomics Analysis

**Figure 1:**
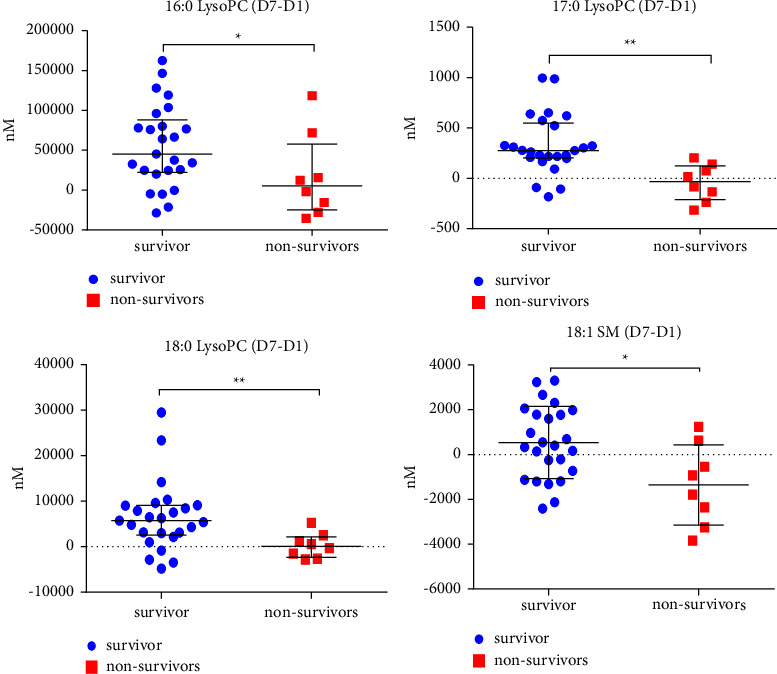
Scatter Plot of D7–D1 lipid levels for the survival and nonsurvival groups. ^∗^ means p < 0.05 ^∗∗^ means p < 0.01.D7–D1 LysoPC level (16 : 0, 17 : 0,18 : 0) and 18 : 1 SM levels were significantly higher in the survivor group than in the nonsurvivor group. D1, day 1; D7, day 7; D7–D1, change between D7 and D1; LysoPC, lysophosphatidylcholine.

In total, 113 lipids were detected in plasma samples on day 1 and day 7, of which 42 lipids were most abundant in plasma samples (Table S1 shows Rt and m/z of 42 kinds of lipids).  Table 2 shows all lipid results in D1, D7, and D7–D1 survival and nonsurvival groups. We found that the difference in lipid concentration between groups was significant for D7-D1.  Figure 1 shows the scatter plot of D7-D1(changes between D7 and D1) differential metabolites between the survival and nonsurvival groups.

### 3.3. Relationship between T Lymphocyte Subsets and Lipids

**Figure 2:**
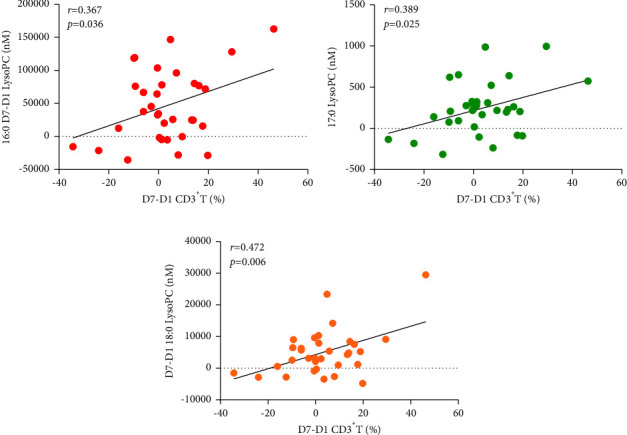
Correlation between D7–D1 plasma lipid levels and D7–D1 lymphocyte subsets. ^*∗*^p  <  0.05 indicated that the difference was statistically significant. (a) D7–D1 CD3^+^T lymphocyte percentage correlates with D7–D1 16 : 0 LysoPC. (b) D7–D1 CD3^+^T lymphocyte percentage correlates with D7–D1 17 : 0 LysoPC. (c) D7–D1 CD3^+^T lymphocyte percentage correlates with D7-D1 18 : 0 LysoPC.

In order to reveal the correlation between changes of lipid metabolism with lymphocyte disturbance in sepsis, we analyzed the lymphocyte subsets on D1 and D7 in survivor and nonsurvivor groups, and found that there were significant differences in CD3^+^ T lymphocytes in the blood on D1 and D7 in both survivor and nonsurvivor groups (Table 3). According to the results in Table 2, the between-group difference in plasma lipid levels was greater for D7–D1 than for D1 or D7. We analyzed the correlation between D7–D1 plasma lipid levels and D7–D1 lymphocyte subsets, and found that D7–D1 plasma 16 : 0 LysoPC (*r* = 0.367, *p*=0.036),17 : 0 LysoPC(*r* = 0.389, *p*=0.025) and 18 : 0 LysoPC(*r* = 0.472, *p*=0.006) levels were positively correlated with the percentage of CD3^+^ T cell in the D7–D1 (Figure 2).

## 4. Discussion

In this study, we investigated differences in lipid metabolism between surviving and nonsurviving patients with sepsis based on their 28-day survival status. There are 42 lipids in plasma, including LysoPC PC, PE, LysoPE LysoPI, SM, and ceramide. Scatter plots were used to represent the above-given differential metabolites (Figure 1), and we found that LysoPC and SM of the D7–D1 survival group were higher than those of the nonsurvival group, and the differences were statistically significant.

LysoPC, a lipid mediator and major phospholipid component, is involved in immune cell recruitment, stimulation, and infection [12–14]. Kamisoglu et al. [9] reported that five LysoPC type levels were significantly lower in patients with sepsis than in healthy controls. In our study, the LysoPC concentrations in the nonsurvivor group decreased significantly by D7. Drobnik et al. [12] demonstrated that the LysoPC level was significantly lower in patients with sepsis than those in healthy people. Moreover, samples obtained from patients with sepsis on days 4 and 7 revealed an association between LysoPC concentrations and sepsis-related mortality. Park et al. [10] noted that serial measurements of LysoPC concentrations predicted 28-day mortality in ICU patients with severe sepsis and septic shock.

The results of this study were largely consistent with those of previous studies. However, previous studies only analyzed on D1 and D7, and our study emphasized the significance of dynamic monitoring. Repeated measurements indicated that the decrease in LysoPC concentration was more evident in the nonsurvivor than that in the survivor group. This decrease may be attributed to the reduced expression of lipopolysaccharide-induced tissue factor in monocytes owing to changes in LysoPC [15], which can attenuate the release of proinflammatory cytokines and promote the release of anti-inflammatory cytokines [16]. The persistently decreased LysoPC levels in patients with sepsis result primarily from disruptions to metabolic homeostasis [17]. In our study, the decreased LysoPC levels in the nonsurvivor group were associated with sepsis-induced immune overreaction, possibly leading to LysoPC consumption. A study [18] using ^1^H nuclear magnetic resonance to evaluate sepsis-induced acute lung injury (ALI) in 13 patients reported that sphingolipid levels were lower in patients with sepsis-induced ALI than in healthy controls. Although the specific mechanisms remain unclear, our results indicate that LysoPC could be a prognostic sepsis biomarker.

SM lipids, including sphingosines, fatty acids, phosphoric acid, and nitrogenous bases, are major membrane constituents [19]. Moreover, SM lipids and their metabolites are essential signaling molecules that regulate key signal transduction processes, including cell growth, differentiation, senescence, and apoptosis [20]. Previous studies have reported that SM is involved in sepsis-induced lung injury [21, 22]. Mecatti et al. [5] performed a lipidomics analysis of plasma and erythrocytes in 20 patients with sepsis, and lower levels of SM, PC, and LysoPC were found in these patients than in healthy controls. Arshad et al. [23] also observed low SM, PC, and LysoPC levels and high acid sphingomyelinase (aSMAse) activity in patients with community-acquired pneumonia, with lipid levels gradually returning to the normal range with clinical improvement. Sepsis involves increases in aSMAse plasma levels and activity, which are correlated with disease severity [24]. The aforementioned studies suggest an association between SM and sepsis severity, possibly due to SM involvement in the inflammatory response. Similarly, we observed a decrease in plasma SM levels in patients with sepsis, which was more apparent in the nonsurvivor than in the survivor group. This difference may be attributed to SM hydrolysis to ceramide, which exerts anti-inflammatory effects by inhibiting reactive oxygen species, mitogen-activated protein kinases, phosphatidylinositol-3-kinase, protein kinase B, Janus kinase/signal transducer, and activators of transcription pathways, while upregulating protein kinase A and heme oxygenase-1 expression [25].

Sepsis is a host response disorder to infection that leads to life-threatening organ dysfunction [11] and reflects the complex response of the host immune system to pathogens [26]. During sepsis, CD4^+^ T cells are activated in response to antigen presentation by dendritic cells or monocytes, releasing immunomodulators and coordinating cytotoxic CD8^+^ T cells [27]. Moreover, low lymphocyte levels and nonrecovery are associated with sepsis mortality risk [28]. LysoPC is produced by the action of phospholipase A2 on PC and promotes inflammatory effects, including endothelial cell adhesion, monocyte chemotactic activation, and macrophage activation [29]. Lin et al. [30] demonstrated that the most abundant plasma LysoPC species (16 : 0, 18 : 0, and 18 : 1) inhibited the production of reactive oxygen species and activation of neutrophils, and LysoPC prevented neutrophil-mediated pulmonary vascular injury in an *in vitro* lung perfusion model. Asaoka et al. [31] found that LysoPC specifically enhanced T lymphocyte activation. Furthermore, LysoPC enhances interferon-*γ* secretion, activates CD4^+^ and CD8^+^ T cells, and increases CD40L and C-X-C chemokine receptor type 4 expression in CD4^+^ T cells [32]. In addition, Ni et al. [33] observed that plasmalogen lysophosphatidylethanolamine was a type of autoantigen that stimulated natural killer T cell production and activation. In our preliminary analysis, the D7–D1 LysoPC (16 : 0, 17 : 0,18 : 0) plasma concentration was positively correlated with the percentage of CD3^+^ T cell in the D7–D1. These results suggest that lipids play a role in the disturbance of lymphocyte homeostasis during sepsis, although further investigation is required.

The aging process is characterized by decreased immune function and increased stress response. Oxidative and metabolic stress may lead to changes in sphingolipid metabolism and increase the risk of age-related diseases. However, an association was reported between age and 18:0–22 : 6 PC lipids in women and between age and eicosapentaenoic acid-containing lipids (e.g., 16:0–20 : 5 PC and 20 : 5 cholesteryl ester) and 18:1–22 : 0 ceramide in men; however, no other lipid values were correlated with age [34]. A study involving 800 healthy volunteers reported no correlation between total LysoPC levels and age [35]. Another study reported that the in-hospital mortality risk of sepsis was significantly associated with advanced age [36]; furthermore, the survivor group had higher BMIs than the nonsurvivor group, although the difference was nonsignificant. While obesity is associated with an inflammatory response, the underlying mechanisms remain unclear. High BMI was previously reported as a protective factor against sepsis [37]. Recently, an inverse association between LysoPCs (17 : 0, 18 : 1, and 18 : 2) and BMI was reported [38]. Bagheri et al. demonstrated that LysoPC (18 : 1 and 18 : 2) values, although not SM values, were negatively associated with obesity in Iranian adults [39]. No such correlations were observed in our study, likely because of differences in the study populations. Further studies are required to clarify whether sepsis differentially affects lipid metabolism in patients with and without obesity.

This study had limitations. It did not include a healthy control group since previous studies have repeatedly demonstrated lipid variations in patients with sepsis. Moreover, given the lack of an external validation cohort and small sample size, our findings require validation.

## 5. Conclusions

In conclusion, our findings indicate that repeated monitoring of lipids in plasma LysoPC and SM may help predict the prognosis of patients with sepsis. Furthermore, the correlation between changes in lipids and lymphocyte subsets suggests that lipid metabolism plays a role in the immune disturbance that occurs during sepsis.

## Figures and Tables

**Table 1:** Clinical characteristics of the participants.

Variable	Survivors (*n* = 25)	Nonsurvivors (*n* = 15)	*p*-value
Age, years	78 (52–84)	83 (76–85)	0.105
Men	12 (48)	12 (80)	0.046^*∗*^
BMI (kg/m^2^)	25 (20–29)	22 (19–28)	0.224
Primary site of infection			
Lung	20 (80)	11 (73.3)	0.922
Other site	5(20)	4 (27)	0.922
Comorbidities			
COPD	1 (4)	1 (6.67)	1.000
DM	9 (36)	3 (20)	0.476
Hypertension	13 (52)	10 (66.7)	0.364
Cerebrovascular disease	12 (48)	6 (40)	0.622
ARDS	13 (52)	10 (67)	0.364
Duration of MV (days)	6 (2–10)	7 (2–14)	0.594
Length of hospital stay (days)	17 (12–25)	15 (6–20)	0.088

Values are expressed as *n* (%) or median (25%–75%) unless otherwise stated. BMI, body mass index; COPD, chronic obstructive pulmonary disease; DM, diabetes mellitus; ARDS, acute respiratory distress syndrome; MV, mechanical ventilation.

**Table 2:** Plasma lipid data of survivor and nonsurvivor groups.

Variable (nM)	D1			D7			D7-D1		
	Survivors (*n* = 25)	Nonsurvivors (*n* = 15)	*p*-value	Survivors (*n* = 25)	Nonsurvivors (*n* = 8)	*p*-value	Survivors (*n* = 25)	Nonsurvivors (*n* = 8)	*p*-value
16 : 0 LysoPC	47645.79 (31495.28–89381.93)	54143.53 (24175.21–110959.19)	0.900	117402.86 (76388.19–153896.00)	38892.97 (26863.80–109655.06)	0.017^*∗*^	45344.39 (22450.94–88171.33)	5273.77 (−24878.11–57746.44)	0.048^*∗*^
14 : 0 LysoPC	562.61 (443.30–625.22)	544.81 (387.80–674.52)	0.727	854.10 (691.57–1058.04)	731.14 (515.61–927.74)	0.193	292.74 (98.52–512.49)	89.55 (0.80–163.51)	0.153
15 : 0 LysoPC	566.46 (442.74–668.70)	594.59 (412.87–720.05)	0.769	889.98 (635.55–985.57)	716.97 (553.37–812.88)	0.153	206.08 (75.76–464.47)	83.36 (−14.53–159.54)	0.110
17 : 0 LysoPC	554.96 (380.61–739.33)	517.30 (341.62–855.38)	0.685	858.26 (672.48–1149.97)	574.04 (430.60–686.53)	0.005^*∗*^	275.04 (203.05–549.00)	−32.92 (−211.46–124.50)	0.001^*∗*^
18 : 0 LysoPC	6778.18 (3811.50–10874.18)	7025.67 (2518.06–9248.98)	0.586	13450.31 (9228.13–17360.12)	5259.48 (3231.33–10666.63)	0.004^*∗*^	5718.02 (2554.51–9075.89)	120.06 (−2361.27–2198.87)	0.010^*∗*^
18 : 1 LysoPC	6022.53 (3553.98–8608.39)	6224.57 (2203.90–10340.38)	0.922	12912.09 (8657.25–17139.27)	6056.01 (2494.34–14707.32)	0.040^*∗*^	5435.26 (1390.30–12651.51)	750.78 (−804.26–8620.02)	0.179
20 : 4 LysoPI	722.37 (445.65–1308.57)	780.50 (451.08–2158.25)	0.349	699.68 (438.84–1293.19)	651.92 (391.67–1176.63)	0.501	−38.40 (−187.64–78.05)	−25.59 (−57.42–344.32)	0.294
18 : 0 PE	1493.12 (1201.51–2745.27)	2280.28 (1445.53–3225.21)	0.167	2142.35 (1595.05–3186.86)	3664.51 (2875.74–6476.39)	0.036^*∗*^	507.00 (139.72–4095.54)	729.71 (−298.53–4177.34)	0.867
16:0–18 : 1 PE	3491.63 (2551.85–5749.08)	6750.93 (3238.72–8458.05)	0.158	3725.58 (2051.79–7734.99)	10951.61 (4649.92–16254.12)	0.019^*∗*^	447.28 (−1202.71–4095.54)	6806.34 (489.33–9295.82)	0.059
16:0–18 : 2 PE	5971.65 (4667.43–12012.34)	14213.70 (10082.27–19008.73)	0.026^*∗*^	6335.59 (3940.83–19357.27)	17851.65 (9616.94–41152.45)	0.053	1834.57 (−1665.37–8588.23)	8366.01 (1089.32–21130.06)	0.193
16:0–20 : 4 PE	6264.81 (4058.03–12709.27)	9581.70 (6905.14–13060.32)	0.150	7692.52 (4141.69–14919.92)	12910.23 (7285.30–22963.00)	0.223	734.46 (−1914.44–5549.51)	5083.14 (−955.67–8134.60)	0.334
16:0–22 : 6 PE	19348.42 (12947.13–46886.62)	37492.20 (23092.63–50996.44)	0.214	28178.89 (13008.68–59176.73)	59446.43 (22453.56–73603.91)	0.223	5614.04 (−5768.61–35450.20)	22605.58 (4309.01–38569.88)	0.257
18:0–18 : 2 PE	12733.97 (10594.57–20815.69)	23814.87 (20595.80–46621.78)	0.017^*∗*^	15494.19 (10115.09–33258.62)	38422.61 (20754.93–59676.60)	0.053	3390.22 (−1419.69–14017.09)	5849.92 (−3689.90–34346.29)	0.737
18:0–20 : 4 PE	195308.16 (144022.72–316482.95)	322636.03 (234243.32–401924.15)	0.142	248414.01 (120406.60–394477.35)	343228.72 (185468.10–448939.29)	0.294	3271.57 (−48274.87–173536.48)	21496.88 (−60103.70–115631.77)	0.933
18:0–22 : 6 PE	7224.19 (5100.28–16136.85)	11880.36 (4813.88–16899.79)	0.426	9161.84 (4877.97–18786.07)	17835.56 (12013.79–22761.61)	0.141	1937.65 (−2091.67–11920.19)	6545.06 (803.73–10169.62)	0.501
14 : 0 LysoPE	555.99 (334.27–3484.52)	353.11 (259.18–1532.81)	0.476	443.99 (193.15–2703.23)	2742.10 (456.81–13156.82)	0.101	−119.46 (−405.53–513.47)	1835.86 (−78.69–8644.08)	0.085
16 : 0 LysoPE	4453.28 (2364.01–8896.05)	5100.30 (2747.04–9349.86)	0.856	8210.14 (4510.54–10939.53)	8208.28 (5078.70–10758.13)	0.966	3461.40 (703.63–7092.41)	2596.76 (1621.38–5808.26)	0.900
18 : 0 LysoPE	1826.52 (1413.53–3211.28)	1809.18 (1016.44–3238.54)	0.511	3826.56 (2331.47–4850.50)	2180.73 (1250.18–3159.32)	0.036^*∗*^	1006.70 (17.66–2485.23)	605.07 (−270.03–1262.71)	0.425
18 : 1 LysoPE	2097.79 (939.33–4891.90)	2431.32 (653.89–4078.73)	0.944	3360.97 (2614.74–5520.26)	5256.52 (2591.47–9697.28)	0.529	1998.87 (−399.72–2639.20)	1542.73 (288.15–7518.27)	0.834
15 : 0 PC	4085.07 (2926.55–5553.55)	4349.65 (2461.94–5276.59)	0.989	3801.10 (2327.55–5570.09)	4288.43 (2942.47–6122.28)	0.674	−131.06 (−1102.35–1590.94)	1229.49 (−1355.13–1870.44)	0.401
16 : 0 PC	29703.76 (25474.95–42230.58)	35894.33 (22077.55–48070.41)	0.494	30432.44 (24713.70–40188.12)	30571.81 (20737.63–36444.16)	0.585	609.53 (−4776.65–4269.97)	−2842.23 (−9211.40–1481.85)	0.355
17 : 0 PC	22393.56 (6104.38–39570.25)	31940.92 (4004.49–44439.10)	0.605	25331.06 (7159.97–42717.74)	22169.66 (3396.93–54568.61)	0.705	871.32 (−2114.08–3755.21)	601.81 (−118.18–11814.62)	0.585
16 : 1 (Δ9–Cis) PC	2035.07 (1139.23–3367.16)	2219.49 (1406.17–4142.06)	0.364	2347.80 (1563.95–4028.63)	2473.91 (1485.48–5075.80)	0.933	725.71 (−297.81–1478.72)	499.71 (−226.39–1347.20)	0.644
18 : 1 (Δ6–Cis) PC	261178.44 (195560.70–355721.48)	339413.93 (2493.18–443771.49)	0.150	329095.33 (235899.83–400610.19)	221229.71 (184490.45–391672.19)	0.257	56856.96 (−50362.91–129076.83)	−62696.57 (−257199.48–2990.53)	0.017^*∗*^
18 : 2 (Cis) PC (DLPC)	184199.14 (13610.08–301147.62)	202776.66 (29317.93–379207.71)	0.459	176871.95 (25541.39–301606.26)	59002.20 (10548.48–240050.72)	0.193	−267.98 (−34042.76–14970.49)	325.01 (−49320.78–43124.56)	0.933
20 : 4 (CisPC	950.81 (711.96–1284.71)	1017.86 (880.51–1352.09)	0.748	1291.29 (1074.31–1633.81)	764.50 (344.66–984.90)	0.001^*∗*^	359.83 (−64.11–658.44)	−9.08 (−134.27–43.44)	0.032^*∗*^
16:0–18 : 1 PC	367968.93 (293044.87–478156.82)	407921.85 (339975.21–611837.27)	0.258	393292.77 (244105.72–609542.74)	503048.65 (373013.97–624819.66)	0.334	59498.46 (−68655.93–183371.08)	58029.25 (30467.89–201823.68)	0.450
18:0–22 : 6 PC	12707.22 (8572.14–15919.73)	11393.47 (7586.36–14668.03)	0.426	12586.54 (7885.99–17052.51)	9246.98 (3893.92–11631.45)	0.032^*∗*^	−424.36 (−3051.63–5472.86)	−3238.85 (−5093.89–182.75)	0.093
18:1–18 : 0 PC	30919.73 (24618.95–39336.74)	31332.57 (27753.23–43794.64)	0.567	46915.50 (34774.84–61301.82)	48831.19 (37985.70–54468.56)	0.769	7093.81 (1054.35–28166.38)	11643.09 (−876.27–18644.37)	0.737
16 : 0 SM	240873.95 (76481.33–311554.44)	305080.43 (85113.52–361243.22)	0.194	238174.68 (76941.20–334210.42)	131647.18 (43982.20–223070.37)	0.053	−8772.97 (−49228.81–22084.70)	−23679.46 (−36682.05–−7178.98)	0.401
17 : 0 SM	3509.81 (2278.21–4340.59)	4147.07 (2107.43–5222.32)	0.335	3581.18 (1931.35–4417.85)	2358.71 (985.82–3560.52)	0.110	−57.00 (−841.94–441.83)	−569.16 (−724.59–70.64)	0.208
18 : 0 SM	36630.75 (18728.18–48618.78)	34702.66 (16191.61–56476.49)	0.685	28706.78 (15548.77–55309.55)	16192.68 (5140.87–33252.71)	0.110	−3066.21 (−8039.50–6457.16)	−4126.86 (−6741.35–392.36)	0.867
18 : 1 SM	2868.66 (2164.78–5085.49)	3437.40 (2846.27–4393.62)	0.349	4009.61 (2562.18–5334.50)	3056.94 (2224.28–3962.18)	0.141	392.19 (−930.29–1880.62)	−1358.22 (−3028.30–328.19)	0.023^*∗*^
24 : 0 SM	17360.63 (11729.22–34205.97)	30746.35 (15078.40–38704.38)	0.185	20905.22 (12342.47–32776.33)	19001.57 (5103.74–28461.75)	0.378	70.05 (−5114.18–5536.13)	−4431.31 (−5523.38–8206.74)	0.401
24 : 1 SM	77329.53 (48739.03–150692.35)	120008.44 (56023.29–171201.33)	0.235	116073.97 (58571.47–165057.54)	68616.14 (34768.06–154764.03)	0.257	5636.92 (−17507.22–29374.29)	−11454.39 (−25321.07–60237.72)	0.401
16 : 0 ceramide	2934.78 (1677.44–4628.57)	4020.45 (1231.47–5189.63)	0.769	2661.30 (1284.48–3143.01)	2554.08 (906.93–4054.96)	0.801	−641.42 (−1570.97–71.92)	−575.98 (−1272.81–−137.78)	0.966
18 : 0 ceramide	2001.39 (1061.58–3242.56)	2212.33 (782.03–3023.55)	0.791	1780.69 (1082.96–2499.12)	1697.29 (720.56–3060.19)	0.933	−216.07 (−844.71–282.25)	−93.96 (−1404.07–360.25)	0.867
18 : 1 ceramide	911.56 (550.07–1300.82)	1006.46 (536.90–1338.42)	0.878	893.39 (549.48–1121.13)	786.71 (526.98–1207.20)	0.769	−29.78 (−201.78–21.02)	−8.96 (−243.81–19.14)	0.674
20 : 0 ceramide	1658.83 (986.15–2092.05)	1764.86 (920.03–2798.69)	0.379	1355.39 (950.57–1830.90)	1306.95 (743.49–2167.06)	0.501	−109.40 (−422.72–158.68)	−134.37 (−1159.48–−18.95)	0.179
22 : 0 ceramide	304.84 (267.70–443.87)	337.13 (296.71–530.06)	0.175	362.14 (254.03–527.38)	376.95 (300.57–574.25)	0.585	−19.40 (−84.89–201.51)	−34.03 (−164.26–251.51)	0.556
24 : 0 ceramide	31362.79 (25605.08–42431.63)	30447.84 (20865.83–45588.97)	0.922	29859.38 (24106.03–40325.47)	35167.62 (22076.35–45854.22)	0.475	−1021.46 (−8034.04–11936.68)	2016.01 (−5911.50–5192.63)	0.801
24 : 1 ceramide	5527.79 (3948.79–7456.97)	6419.72 (3379.74–11735.65)	0.158	4532.09 (3229.22–7040.63)	4173.49 (1575.18–10255.01)	0.674	174.54 (−1881.11–1654.20)	−418.20 (−3031.20–912.74)	0.529

*P*  <  0.05 was considered statistically significant. D1, day 1; D7, day 7; DLPC, dioleoylphosphatidylcholine; PC, phosphatidylcholine; PE, phosphatidylethanolamines; PI, phosphatidylinositol; SM, sphingomyelin.

**Table 3:** Results of D1 and D7 immune cell subsets in sepsis patients.

Variable (%)	Survivors (*n* = 25)	Nonsurvivors (*n* = 15)	*p-*value

*D1*			
CD3^+^T	66.68 (60.73–72.89)	46.75 (41.92–62.88)	0.007^*∗*^
CD4^+^T	57.48 (45.25–66.51)	66.77 (56.28–71.22)	0.093
CD8^+^T	38.58 (27.22–48.92)	27.64 (23.87–34.99)	0.081
NKT	6.77 (3.48–9.65)	3.28 (2.14–6.28)	0.028^*∗*^
NK	18.52 (11.79–32.85)	24.99 (15.55–36.18)	0.426
B	9.16 (5.09–18.69)	13.72 (8.04–32.46)	0.166
Neu	83.89 (68.46–87.61)	85.94 (74.54–93.37)	0.361
Monocyte	3.92 (2.83–6.26)	3.94 (1.82–6.79)	0.952
MDSCs	0.51 (0.17–3.65)	0.95 (0.34–4.17)	0.469

*D7*	*Survivors* (*n* *=* *25)*	*Non-survivors* (*n* *=* *8)*	*p-value*

CD3^+^T	72.23 (61.91–77.52)	47.40 (35.90–58.93)	0.000^*∗*^
CD4^+^T	57.74 (50.80–62.87)	48.23 (31.83–72.79)	0.298
CD8^+^T	37.63 (23.85–42.07)	37.20 (20.43–48.17)	0.853
NKT	7.38 (3.84–10.27)	5.53 (2.92–7.26)	0.334
NK	15.87 (8.99–22.31)	20.47 (13.16–35.76)	0.176
B	8.14 (5.00–16.44)	13.73 (5.07–35.23)	0.352
Neu	79.38 (66.92–84.84)	89.58 (76.13–92.94)	0.074
Monocyte	3.68 (2.10–5.95)	4.03 (1.86–5.48)	1.000
MDSCs	0.72 (0.21–3.16)	0.17 (0.11–4.22)	0.344

Values are expressed as *n* (%) or median (25%–75%) unless otherwise stated.

## Data Availability

The datasets used and/or analyzed during the current study are available from the corresponding author on reasonable request.

## Supplementary Materials

Supplementary Materials All the measured lipids (incl Rt and measured ion (m/z).
