# Supplementary material for: Association between Changes in Plasma Metabolism and Clinical Outcomes of Sepsis
Source: Emerg Med Int. 2023 Jun 13;2023:2590115. doi: 10.1155/2023/2590115 (PMC10281824; doi:10.1155/2023/2590115)
Supplement: Supplementary Materials — All the measured lipids (incl Rt and measured ion (m/z). [file 2590115.f1.docx]

Table S1 All the lipids Rt and m/z

| **Number** | **Name** | **Molecular** | **MS** | **M/Z** | **RT** |
| --- | --- | --- | --- | --- | --- |
| 1 | 16:0 LysoPC | C_24_H_50_NO_7_P | 495.33 | 540.3296 | 3.21 |
| 2 | 14:0 LysoPC | C_22_H_46_NO_7_P | 467.301 | 512.2983 | 2.72 |
| 3 | 15:0 LysoPC | C_23_H_48_NO_7_P | 481.317 | 526.31395 | 2.94 |
| 4 | 17:0 LysoPC | C_25_H_52_NO_7_P | 509.348 | 554.34525 | 3.53 |
| 5 | 18:0 LysoPC | C_26_H_54_NO_7_P | 523.364 | 568.3609 | 3.88 |
| 6 | 18:1 LysoPC | C_26_H_52_NO_7_P | 521.348 | 566.34525 | 3.28 |
| 7 | 20:4 Lyso PI | C_29_H_52_NO_12_P | 637.323 | 619.28779 | 2.65 |
| 8 | 18:0 PE | C_41_H_82_NO_8_P | 747.578 | 746.57053 | 8.67 |
| 9 | 16:0-18:1 PE | C_39_H_76_NO_8_P | 717.531 | 716.52358 | 8.18 |
| 10 | 16:0-18:2 PE | C_39_H_74_NO_8_P | 715.515 | 714.50793 | 7.59 |
| 11 | 16:0-20:4 PE | C_41_H_74_NO_8_P | 739.515 | 738.50793 | 7.39 |
| 12 | 16:0-22:6 PE | C_43_H_74_NO_8_P | 763.515 | 762.50793 | 7.13 |
| 13 | 18:0-18:2 PE | C_41_H_78_NO_8_P | 743.547 | 742.53923 | 8.25 |
| 14 | 18:0-20:4 PE | C_43_H_78_NO_8_P | 767.547 | 766.53923 | 8.05 |
| 15 | 18:0-22:6 PE | C_45_H_78_NO_8_P | 791.547 | 790.53923 | 7.79 |
| 16 | 14:0 Lyso PE | C_19_H_40_NO_7_P | 425.254 | 424.24697 | 2.78 |
| 17 | 16:0 Lyso PE | C_21_H_44_NO_7_P | 453.286 | 452.27827 | 3.29 |
| 18 | 18:0 Lyso PE | C_23_H_48_NO_7_P | 481.317 | 480.30957 | 3.98 |
| 19 | 18:1 Lyso PE | C_23_H_46_NO_7_P | 479.301 | 478.29392 | 3.36 |
| 20 | 15:0 PC | C_38_H_76_NO_8_P | 705.531 | 750.52796 | 7.32 |
| 21 | 16:0 PC (DPPC) | C_40_H_80_NO_8_P | 733.562 | 778.55926 | 8.04 |
| 22 | 17:0 PC | C_42_H_84_NO_8_P | 761.593 | 806.59056 | 8.67 |
| 23 | 16:1 (Δ9-Cis) PC | C_40_H_76_NO_8_P | 729.531 | 774.52796 | 6.86 |
| 24 | 18:1 (Δ6-Cis) PC | C_44_H_84_NO_8_P | 785.593 | 830.59056 | 8.22 |
| 25 | 18:2 (Cis) PC (DLPC) | C_44_H_80_NO_8_P | 781.562 | 826.55926 | 6.8 |
| 26 | 20:4 (Cis) PC | C_48_H_80_NO_8_P | 829.562 | 874.55926 | 6.41 |
| 27 | 16:0-18:1 PC | C_42_H_82_NO_8_P | 759.578 | 804.57491 | 8.01 |
| 28 | 18:0-22:6 PC | C_48_H_84_NO_8_P | 833.593 | 878.59056 | 7.64 |
| 29 | 18:1-18:0 PC | C_44_H_86_NO_8_P | 787.609 | 832.60621 | 8.61 |
| 30 | 16:0 SM | C_39_H_79_N_2_O_6_P | 702.568 | 747.56468 | 7.42 |
| 31 | 17:0 SM | C_40_H_81_N_2_O_6_P | 716.583 | 761.58033 | 7.81 |
| 32 | 18:0 SM | C_41_H_83_N_2_O_6_P | 730.599 | 775.59598 | 8.19 |
| 33 | 18:1 SM | C_41_H_81_N_2_O_6_P | 728.583 | 773.58033 | 7.43 |
| 34 | 24:0 SM | C_47_H_95_N_2_O_6_P | 814.693 | 859.68988 | 9.91 |
| 35 | 24:1 SM | C_47_H_93_N_2_O_6_P | 812.677 | 857.67423 | 9.28 |
| 36 | 16:0 ceramide | C34H67NO3 | 537.5121 | 582.5092 | 7.96 |
| 37 | 18:0 ceramide | C36H71NO3 | 565.5434 | 610.5405 | 8.62 |
| 38 | 18:1 ceramide | C36H69NO3 | 563.52775 | 608.5249 | 7.96 |
| 39 | 20:0 ceramide | C38H75NO3 | 593.5747 | 638.5718 | 9.2 |
| 40 | 22:0 ceramide | C40H79NO3 | 621.606 | 666.6031 | 9.7 |
| 41 | 24:0 ceramide | C42H83NO3 | 649.6373 | 694.6344 | 10.17 |
| 42 | 24:1 ceramide | C42H81NO3 | 647.62165 | 692.6188 | 9.6 |
